# Assessing the relative importance of bacterial resistance, persistence and hyper-mutation for antibiotic treatment failure

**DOI:** 10.1098/rspb.2022.1300

**Published:** 2022-11-09

**Authors:** Christopher Witzany, Roland R. Regoes, Claudia Igler

**Affiliations:** Institute of Integrative Biology, ETH Zurich, Zurich, Switzerland

**Keywords:** antibiotic resistance evolution, persisters, hyper-mutators, stochastic simulations, pharmacodynamic modelling, fitness calculations

## Abstract

To curb the rising threat of antimicrobial resistance, we need to understand the routes to antimicrobial treatment failure. Bacteria can survive treatment by using both genetic and phenotypic mechanisms to diminish the effect of antimicrobials. We assemble empirical data showing that, for example, *Pseudomonas aeruginosa* infections frequently contain persisters, transiently non-growing cells unaffected by antibiotics (AB) and hyper-mutators, mutants with elevated mutation rates, and thus higher probability of genetic resistance emergence. Resistance, persistence and hyper-mutation dynamics are difficult to disentangle experimentally. Hence, we use stochastic population modelling and deterministic fitness calculations to investigate the relative importance of genetic and phenotypic mechanisms for immediate treatment failure and establishment of prolonged, chronic infections. We find that persistence causes ‘hidden’ treatment failure with very low cell numbers if antimicrobial concentrations prevent growth of genetically resistant cells. Persister cells can regrow after treatment is discontinued and allow for resistance evolution in the absence of AB. This leads to different mutational routes during treatment and relapse of an infection. By contrast, hyper-mutation facilitates resistance evolution during treatment, but rarely contributes to treatment failure. Our findings highlight the time and concentration dependence of different bacterial mechanisms to escape AB killing, which should be considered when designing ‘failure-proof’ treatments.

## 1. Introduction

The evolution of antimicrobial resistance is a global and growing threat to human lives and contemporary medicine [1]. Studies have shown that the efficacy of antibiotic (AB) treatments is threatened by genetic resistance as well as phenotypic mechanisms, such as persistence and hyper-mutation, which can facilitate resistance evolution or provide other means of escape from ABs [2–5]. To ensure prolonged efficacy of current and future ABs, it is crucial to investigate how genetic and phenotypic mechanisms can emerge and lead to treatment failure.

**Figure 1.**
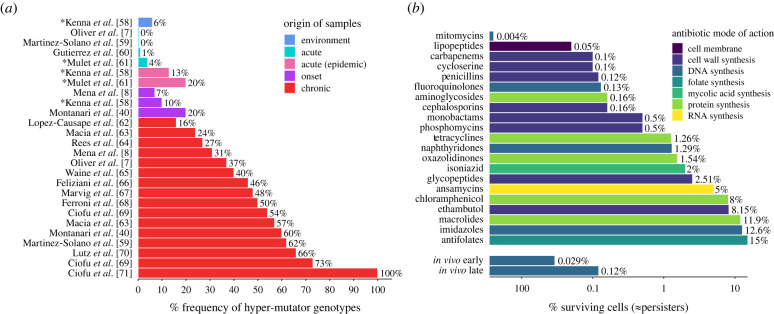
Hyper-mutator and persister frequencies increase over the duration of the infection. (*a*) Hyper-mutator frequencies of *Pseudomonas aeruginosa* (%) compiled from empirical studies. Shown are frequencies for samples from the environment or from patients with early (acute) infection, epidemic infection, onset of prolonged (chronic) infection and chronic infection. For studies marked with an asterisk (*) isolate level data are shown, as patient level data were not available (electronic supplementary material, text S1). (*b*) Persister numbers of multiple species as surviving cells (in %) after exposure to different AB classes (colour-coded according to the mode of action) from *in vitro* and *in vivo* studies assembled from the literature by Salcedo-Sora & Kell [14]. For comparison with clinical data, *in vivo* persister numbers from isolates from early and later infection stages with *P. aeruginosa* are shown separately (data from [15]) (see electronic supplementary material, text S1). (online version in colour.)

In long-lasting infections, such as those caused by *Pseudomonas aeruginosa* or *Mycobacterium tuberculosis*, random chromosomal mutations can emerge over the course of treatment and cause complications or treatment failure [6,7]. The speed by which mutations arise is crucial for pathogen survival. This mutation rate can be increased about 100- to 1000-fold [8,9] through mutations that lead to error-prone replication in so-called hyper-mutators. While most mutations will be deleterious, hyper-mutators are known to flourish in highly fluctuating environments by acquiring beneficial mutations, like AB resistance, that outweigh the cost of deleterious mutations [8,10,11]. Empirical studies with *P. aeruginosa*, *Escherichia coli* [12] and *Staphylococcus aureus* [13] suggest that hyper-mutators are frequently found in clinical settings and increase in number with the duration of an infection (figure 1*a*, electronic supplementary material, text S1). As hyper-mutators significantly increase the probability of resistance emergence (electronic supplementary material, figure S1*a*), their frequent occurrence threatens the efficacy of ABs.

While the emergence of genetic resistance is still considered the main cause of treatment failure, it is becoming increasingly clear that non-genetic mechanisms also enable bacteria to survive AB treatment. One such mechanism is persistence, which describes a phenotypic state, defined by the formation of transiently non-growing bacterial subpopulations that are refractory, i.e. unaffected by ABs [16]. This can be observed as a biphasic killing curve in the presence of ABs (electronic supplementary material, figure S1*b*), where the growing population dies rapidly, leaving the smaller persister population, which declines at a much slower rate.

Persistence has been reported for most AB classes at substantial numbers (figure 1*b*; electronic supplementary material, text S1). Moreover, high-persister mutants can generate larger persister subpopulations than the wild-type [17–19]. High-persistence, and persistence in general, is beneficial in highly fluctuating environments [20,21] and frequently found in infections with *E. coli* [22], *Candida albicans* [23] and *P. aeruginosa* [24]. Notably, similar to hyper-mutators, persister subpopulations and high-persister mutants increase with the duration of infections (figure 1*b*, [15]). Therefore, (high-)persisters could cause the ‘paradox of chronic infections', which describes persistent infections of genetically susceptible pathogens [25]. The prolonged survival of susceptible bacteria due to persistence could also facilitate resistance evolution, by increasing the opportunities for mutations to occur [2], without changing the mutation rate itself.

The frequent occurrence of hyper-mutators and high-persisters in pathogen infections, even in the presence of resistance evolution, suggests that genetic and phenotypic mechanisms are both beneficial for survival in changing environments (such as AB treatment). Further, hyper-mutation and high-persistence mutations might combine into one mutator–persister genotype, as has been found in clinical strains of *P. aeruginosa* [15]. Interestingly, Mulcahy *et al.* [15] found mutator–persisters to be also genetically resistant against ABs, indicating that resistance can still be beneficial, and potentially facilitated by the presence of both hyper-mutation and high-persistence.

Disentangling the contributions and timing of genetic and phenotypic mechanisms leading to treatment failure poses several challenges. Examining the dynamics of (high-)persistence is inherently difficult experimentally due to the stochastic and phenotypic nature of this trait [16,26]. Including hyper-mutators will likely aggravate this problem, due to the high diversity of mutations that can arise and the corresponding necessity for more experimental replicates [27]. Instead, we use a combined pharmacokinetic (describing AB concentration changes over time) and pharmacodynamic (describing the effect of the AB concentration on the pathogen population) model, which is the main class of population models used to predict drug action and pathogen response [28–30]. Previous studies with pharmacokinetic–pharmacodynamic models have demonstrated their success in predicting pathogen evolution and therapy outcome [31–34]. Here, we use them to investigate (i) how mutant populations of hyper-mutators (*M*), high-persisters (*P*) and resistant (*R*) cells—as well as all their respective combinations—evolve during or following AB treatment, (ii) how they affect treatment outcome and explain the simulation outputs by (iii) deriving analytical calculations for the fitness of specific genotypes. We show that *R*, *M* and *P* populations cause or facilitate treatment failure at distinct AB concentrations, infection timescales and final cell numbers. Our goal is not to make precise quantitative predictions, but rather use mathematical modelling to explore under which treatment conditions these populations can emerge and rise in frequency.

## 2. Materials and methods

### (a) Stochastic population model

**Figure 2.**
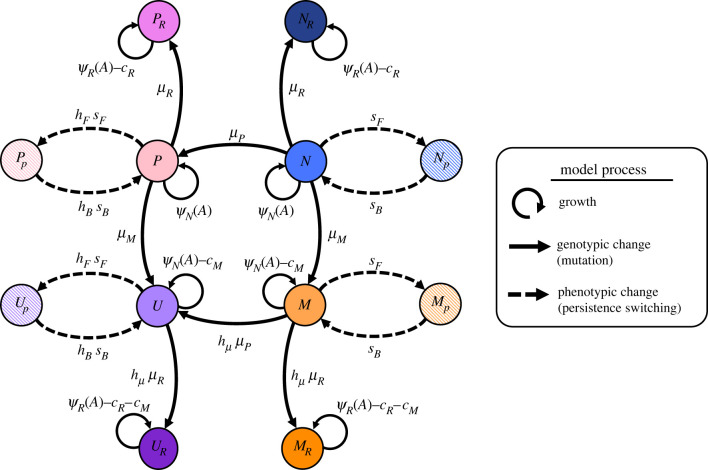
Illustration of the full mathematical model describing resistance, high-persistence and hyper-mutator dynamics. The eight genotypes consist of the wild-type (*N*), hyper-mutators (*M*), high-persisters (*P*), mutator–persisters (*U*) and their corresponding resistant mutants denoted by subscript *R* (electronic supplementary material, table S1). Persister phenotype states (hatched) are denoted by a subscript *p*. Switching between these two states (dashed arrows) happens at rates *s*_F_ and *s*_B_ for *N* and *M*, and with *h*_F_- and *h*_B_-fold increase for *P* and *U*. Net growth rates as determined by AB sensitivity (equation (2.3), electronic supplementary material, text S3) are given as *ψ*_N_ for susceptible (MIC = 1) and as *ψ*_R_ (MIC_R_ = 10 x MIC) for resistant genotypes, together with growth costs due to resistance, *c*_R_, and due to hyper-mutation, *c*_M_. Solid arrows show mutational transitions between genotypes, which happen at rates *µ*_M_, *µ*_P_ and *µ*_R_. Mutators (*M*, *U*) have *h*_µ_-fold increased mutation rates. See electronic supplementary material, table S2 for parameter values.

We investigate the relative importance of high-persistence, hyper-mutation and resistance mutations (leading to *P*, *M* and *R* subpopulations, respectively)—and all their combinations—over the course and after discontinuation of AB treatment by using a stochastic population model (figure 2; electronic supplementary material, table S1). Our model incorporates pharmacokinetic and pharmacodynamic functions to realistically simulate AB treatment (equation (2.1)) and the distinct effects of ABs on our respective subpopulations. As proposed by Balaban *et al.* [17], we describe phenotypic persistence as a two-subpopulation process. For each of our AB-susceptible genotypes (the wild-type *N*, *M*, *P* and mutator–persisters *U*), we model a growing subpopulation, which is affected by ABs (equation (2.3)), and a non-growing (i.e. $\psi_{\max}=0\;$) persister subpopulation, that is unaffected by ABs. The transitions between growing and persister subpopulation happen stochastically at rates $s_{F}\;(N\to N_{p})$ and $s_{B}\;(N_{p}\to N)$ for *N* and *M*, or $h_{F}s_{F}\;(P\to P_{p})$ and $h_{B}s_{B}\;(P_{p}\to P)$ for *P* and *U*. In our model, switching rates are constant (i.e. not environment-dependent) and we assume that resistant subpopulations (*N_R_*, *P_R_*, *M_R_* and *U_R_*) do not generate persisters, as we found that persistence does not convey any additional benefit to already resistant bacteria (electronic supplementary material, figure S2).

For our starting conditions, we assume that only the susceptible wild-type genotype, consisting of its two phenotypic states, *N* and *N_p_*, is present at 1 × 10^9^ colony forming units (CFU) total (*N* + *N_p_*), with *N* and *N_p_* being in equilibrium, according to their respective switching rates. The ratio $N/N_{p}$ at equilibrium is obtained by determining the dominant eigenvector of the analytical solution of the two-population persister model from Balaban *et al.* [17] in the absence of ABs and with our respective parameters (see also [5]).

Growth of the non-persister populations is limited by the overall carrying capacity *K*, which, together with a constant natural death rate *d*, results in realistic competition between genotypes. Since our mutations are coupled to growth, the death rate also enables mutations to occur after capacity is reached. *P* and *M* mutants can only arise by mutation from the susceptible growing *N* population at rates $\mu_{P}\;$and $\mu_{M}$, respectively, and *U* from the susceptible growing *P* and *M* populations at rates $\mu_{P}$ and $\mu_{M}$, respectively. To investigate the relative contribution of *M* and *P* to the emergence of *U*, we separately quantify mutation events from *M* and *P* leading to *U* when mutation to *U* is only possible from either *M* or *P*.

Resistant mutants *N_R_* and *P_R_* emerge via growth-dependent mutation from *N* and *P* at mutation rate $\mu_{R}$, whereas *M_R_* and *U_R_* have elevated mutation rates and arise from *M* and *U* at $h_{\mu }\mu_{R}$. The maximal growth rate of hyper-mutator populations (*M*, *M_R_*, *U* and *U_R_*) is reduced by the cost of hyper-mutation (*c_M_*): $\psi_{\max_{M}}=\psi_{\max}(1-c_{M})\;$(electronic supplementary material, text S2). Similarly, the growth of resistant populations is reduced by the cost or resistance (*c_R_*): $\psi_{\max_{R}}=\psi_{\max}(1-c_{R})$. Note that for *M_R_* and *U_R_,* these costs are multiplicative. See electronic supplementary material, text S3 for the corresponding system of ordinary differential equations. We calculate the probability of genotype survival at the end of the treatment as the fraction of simulation runs per AB concentration where the genotype population size is larger than zero. The average number of surviving cells (CFU) per genotype is calculated over all simulation runs where the final total population size is larger than zero.

### (b) Pharmacokinetic and pharmacodynamic functions

We model AB treatment with periodic dosing intervals and exponential AB decay, as shown in the electronic supplementary material, figure S3, by using the pharmacokinetic function

2.1 $$A(t)=\;\underset{n}{\sum }A_{\max}e^{-k(t-(n-1)\tau )},$$

with $n=1,\ldots ,t_{\max}/\tau$ representing the number of dosing events. For our simulations, we model 8 days of daily AB treatment (*t*_max_ = 192 h, τ = 24 h) and examine a broad range of drug concentrations *A*_max_ covering 0–50 × minimal inhibitory concentration (MIC) of the susceptible populations. Note that due to drug decay, the concentration *A*_max_ denotes the peak AB concentration, but will be generally referred to as drug concentration in the main text. The decay parameter *k* is fixed for all our simulations, except stated otherwise (electronic supplementary material, table S2).

The effect of an AB on a bacterial population is defined by the pharmacodynamic function [34,35]:

2.2 $$\psi (A(t))=\psi_{\max}-E(A(t))\;.$$

with

2.3 $$E(A(t))=\;(\psi_{\max}-\psi_{\min})\frac{{\left(A(t)/\mathrm{MIC}\right)}^{\kappa }}{{\left(A(t)/\mathrm{MIC}\right)}^{\kappa }-\;(\psi_{\min}/\psi_{\max})}\;.$$

The parameters $\psi_{\max}$ and $\psi_{\min}$ describe the maximal and minimal net growth rates in the absence $\left(\psi_{\max}=\psi (A=0)\right)$ or the presence of high amounts of ABs $\left(\psi_{\min}=\psi (A\to \infty )\right)$. At AB concentrations equal to their MIC, bacterial populations do not grow (ψ(A=MIC)=0). Resistant populations are assumed to have MIC_R_ = 10 × MIC, meaning that their growth stops at a 10-fold higher AB concentration than for susceptibles. The Hill parameter *κ* determines the steepness of the pharmacodynamic curve described by equation (2.3), which reflects the sensitivity of bacterial growth to AB concentration changes.

Unless stated otherwise, simulations and calculations are for bactericidal AB treatment. Additionally, we assess how evolutionary dynamics change for bacteriostatic ABs, i.e. ABs that arrest cellular growth, which can inhibit mutational emergence. See electronic supplementary material, text S4 for details. See electronic supplementary material, table S2 for all parameter values of bactericidal and bacteriostatic AB simulation treatments as well as for antimicrobial peptide (AMP) pharmacodynamics.

### (c) Deterministic fitness measures

To investigate which populations should be fittest for different values of *A*_max_, we separately calculate long-term growth rates for each genetically unique population as approximate fitness measures. For all resistant populations (*N_R_*, *M_R_*, *P_R_* and *U_R_*), this is achieved by integrating the pharmacodynamic functions (equation (2.3)), which describe the net growth rate plus the natural death rate *d* (electronic supplementary material, equation S6, S9, S12), over one treatment period (0−τ) and dividing by τ to derive the mean growth rate per hour (see electronic supplementary material, text S5 for detailed explanations and electronic supplementary material, text S6 for closed integrals). Note that we assume that population sizes are far from the carrying capacity, which allows us to only consider exponential growth.

### (d) Relapse simulations

High-persisters and hyper-mutators are both prevalent in chronic infections and persisters have been proposed to cause relapse of infection even in the absence of resistance [25]. Hence, in addition to the 8-day AB treatment described above, we also simulate how and when surviving bacterial populations can cause an infection to relapse after AB treatment ends. For these relapse simulations, we only consider treatment outcomes, where treatment failure is not apparent, which we define by the total surviving population size being less than 10^5^ CFU. If greater than 10^5^ cells survive the treatment, we consider that as an apparent, acute treatment failure and do not run a relapse simulation. In clinic reality, due to individual differences in patients and infections, determining such a cut-off is much more complicated and as such beyond the scope of this work. However, 10^5^ CFU is in line with clinical detection limits, for example for the diagnosis of urinary tract infections [36]. The regrowth of these small surviving populations is simulated according to the equations outlined above, but in the absence of AB administration (only considering the decaying, leftover AB from the treatment), until a total bacterial population size of 10^6^ CFU is reached, or alternatively for a maximum time of 10 years. We assume that relapse will only be noticeable at pathogen loads that are an order of magnitude higher than our detection limit of 10^5^ CFU as monitoring of an ongoing infection likely leads to detection of lower bacterial numbers than for a new or relapse infection.

### (e) Implementation

All simulations, analysis and plots were done in R v3.6.0 [37]. Stochastic simulations were implemented via the Gillespie algorithm using the R-package *adaptivetau* [38]. To test the accuracy of our simulation results, we used different tolerance levels for the relative rate changes in step size selection, which did not change our results notably. The stochastic simulations were run 1000 times for each AB concentration (0–50 × MIC at 0.1 steps) for 8 days for acute treatment and 10 years for relapse. The corresponding R scripts are publicly available at https://github.com/ChrisWitzany/mutator-persister-evolution. Analytical solutions of the population models were determined by using Matlab version R2020b [39].

## 3. Results

### (a) Modelling of persistence, mutator and resistance dynamics

**Figure 3.**
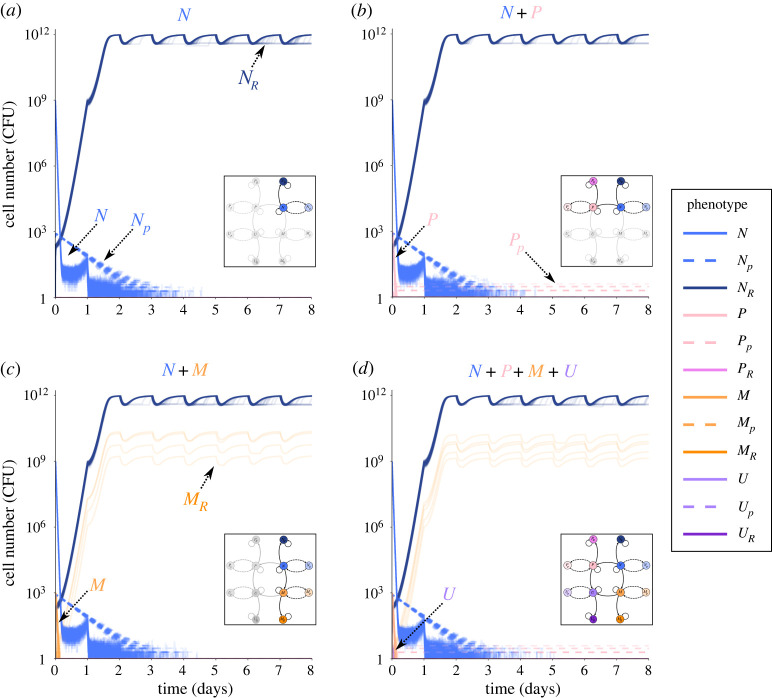
Sub-models of resistant, persistent and hyper-mutator phenotypes. Bacterial numbers (CFU) of various phenotypes obtained by stochastic simulations over an 8-day AB treatment with AB administered at 10 x MIC (= MIC_R_) every 24 h. Each figure shows 100 individual simulation runs. (*a–c*) Population dynamics of the sub-models (as highlighted in the inset). Persister subpopulations are shown as dashed lines, colours of all populations as in figure 2. (*d*) Dynamics of the full model shown in Figure 2.

To investigate the relative importance of phenotypic and genetic mechanisms of bacterial cells to escape AB killing, we use a stochastic pharmacodynamic model (figure 2) to simulate population dynamics during AB treatment (Methods, electronic supplementary material, table S1, text S3). Bacterial persistence can complicate treatment by allowing susceptible bacteria to survive inhibitory AB concentrations, without having to acquire genetic changes [25]. To capture this, we first focus on a submodel that describes the susceptible genotype (*N*), its persister subpopulation (*N_p_*) and a resistant mutant (*N_R_*), which cannot switch into a persister state (figures 2 and 3*a*). *N* and *N_p_* stochastically switch back and forth at rates *s_B_* and *s_F_* (electronic supplementary material, figure S1*b*). We start with *N* and *N_p_* in equilibrium according to the stochastic switching in the absence of AB treatment (Methods), i.e. the growing, AB-sensitive subpopulation *N* at approximately 10^9^ CFU and the non-growing, AB-refractory persisters *N_p_* at approximately 5 × 10^2^ CFU. From the growing subpopulation (*N*), a *de novo* resistant genotype (*N_R_*) with MIC_R_ = 10 × MIC of susceptible cells can arise via random mutation (electronic supplementary material, figure S1*a*). Since we assume that mutations are linked to cellular growth the non-growing persister subpopulation cannot mutate. Hence, *N_p_* can only facilitate resistance emergence through prolonging the survival of the *N* population (electronic supplementary material, figure S3). Note that resistance is, by definition, an increase in MIC, and we assume here that this increase (10 × MIC) is not as large as the highest possible treatment dose (up to 50 × MIC). This is crucial as many single mutations were empirically found to provide relatively small (less than 10 x) increases in MIC (see [33] for references), which is considerably smaller than the maximal clinical dose for many AB types (see https://www.eucast.org/rd).

We simulate 8 days of treatment with an AB dosage equal to MIC_R_ given once every 24 h, which decays over time (electronic supplementary material, table S2). As expected, *N* declines rapidly under treatment and *N_p_* declines at a much slower rate, which is determined by the rate of switching back to *N*, displaying the characteristic biphasic killing curve of persisters ([17], figure 3*a*; electronic supplementary material, figure S1*a*). *N_R_* evolves rapidly from the *N* population and reaches carrying capacity after approximately 2 days. Note that the time until *N_R_* reaches carrying capacity is dependent on the cost of resistance. By contrast, *N* and *N_p_* are fully eradicated after about 4 days. This demonstrates that while persistence prolongs clearance of the susceptible genotype, the emergence of resistance seems to be the sole cause of treatment failure under the simulated regimen. In contrast with our persister simulations (figure 3*a*), treatment failure due to persistence is prevalent in prolonged infections, even in the absence of resistance, but is often linked to the emergence of high-persisters (*P*) [15,21,23,24]. Compared to *N*, *P* cells are mutants which have a higher rate of switching to persistence (*h_F_*-fold) and a lower rate of switching back (*h_B_*-fold ≪ 1), resulting in larger persister fractions with a longer ‘lifespan’. And indeed, if *N* can acquire a high-persistence mutation, the emerging *P* genotype (*P* + *P_p_*) is present at treatment failure at low numbers (figure 3*b*). Specifically, our simulations show that *P* rapidly emerges from *N*, reaches approximately 10^3^ CFU, but then rapidly gets killed by the AB. However, in about half of the cases, a very small fraction of *P* enters the persister state (*P_p_*) before eradication and persists through the whole AB treatment due to the low back-switching rate from *P_p_* (figure 3*b*). Note that resistance could emerge from *P* (*P_R_*), but in most cases, *P* is eradicated before that happens.

High-persisters are not the only problematic mutants increasing in prevalence over the duration of infections [15]. There are also hyper-mutators (*M*), mutants which have an elevated mutation rate, and are associated with facilitated evolution of resistance. However, higher mutation rates come at a growth cost (*c_M_*), due to the accumulation of detrimental mutations [40]. Like with *P*, we can observe the emergence of the *M* genotype (*M* + *M_p_*) from *N* at the beginning of treatment at low frequencies (approx. 10^3^ CFU), but it rapidly gets eradicated (figure 3*c*). In rare cases, *M_R_* evolves before *M* is fully eradicated (approx. 4%). Although *M_R_* and *N_R_* are both able to grow under the applied AB dose, due to *M_R_* emerging at a later point in the treatment and growing slower than *N_R_* by the cost *c_M_*, *M_R_* only reaches population sizes of around 10^9^ CFU, whereas *N_R_* reaches capacity (10^12^ CFU).

Overall, we find that the emergence of the *N_R_* genotype is the predominant cause of treatment failure in this regimen, but that the *P* or *M_R_* genotypes can also survive treatment in approximately 43% and approximately 4% of the cases, respectively. This shows that both *P* and *M* genotypes can confer fitness advantages to *N* cells and complicate treatment. These results are in line with hyper-mutator frequencies in early (acute) infections, which we compiled from the literature (figure 1*a*; electronic supplementary material, text S1), but there is—to our knowledge—no data on high-persister frequencies for acute infections.

Mutations conferring *M* and *P* phenotypes do not have to occur independently. As hyper-mutation is by itself detrimental, but is known to facilitate beneficial mutations other than AB resistance [41], they could acquire the beneficial high-persistence mutation, which would lead to a higher frequency of the combined genotype than that of hyper-mutators alone. Consequently, we allow for the emergence of a combined mutator–persister genotype (*U* + *U_p_*), which has both an increased mutation rate as well as high-persistence switching rates and can evolve from *P* or *M* populations through mutation (figure 2). Further, *U* can acquire a resistance mutation leading to *U_R_*. Interestingly, the population dynamics of the full model (figure 3*d*) reflect the results of the partial models described above: *M* and *P* come up early in the treatment but are quickly eradicated by the AB. *U* also emerges at the beginning of treatment but reaches even lower population sizes than *M* or *P* and is quickly wiped out. The only viable cells left at the end of the 8-day treatment period belong mainly to *N_R_*, with low numbers of *M_R_* and *P_p_* populations surviving as well.

### (b) Resistance and high-persistence cause treatment failure at distinct antibiotic concentrations

**Figure 4.**
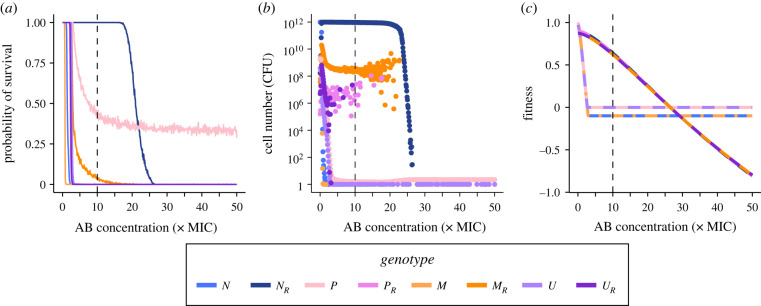
Treatment failure due to wild-type or mutant genotypes. Effect of AB concentration on (*a*) the probability of genotype survival at the end of treatment over 1000 simulations runs (see electronic supplementary material, figure S4 for corresponding probability of overall treatment failure), (*b*) the mean absolute cell numbers (CFU) of surviving genotypes and (*c*) deterministic fitness values of the genotypes. (*a*,*b*) The endpoints of stochastic simulation runs for 8-day AB treatments at various concentrations (0–50 × MIC), starting from a population of wild-type cells (*N* + *N_p_*). Fitness of non-resistant populations in (*c*) reflects a combination of the underlying growing and non-growing (i.e. persister) subpopulations. Note that in (*c*), the fitness curve for *N_R_* (*P_R_*) largely overlaps with the fitness curve for *M_R_* (*U_R_*) for AB concentrations greater than 10 × MIC. The vertical dashed line shows MIC_R_.

So far, we only considered one AB concentration (10 × MIC), which corresponds to the MIC of the *R* populations (MIC_R_), but the fitness of the various subpopulations depends on the strength of the selection pressure due to AB. Hence, we investigated which mutant genotype is expected to emerge and establish itself under different AB concentrations. To examine this, we used the full model to determine the probability of a genotype to survive 8 days of treatment for a range of AB concentrations (0–50 × MIC). Our simulations show that for sub-MIC survival of all genotypes is possible but starts declining at different concentrations greater than MIC (figure 4*a*; electronic supplementary material, figure S4). The first genotype to reach zero probability of survival is *M*, reflecting that hypermutability is costly and without immediate benefit. Then the probability for *N* survival drops to zero, followed by that of the resistant genotypes *P_R_* and *U_R_*, which already declines for AB concentrations greater than 0.5 × MIC_R_, whereas *M_R_* survival only reaches zero at about twice the MIC_R_. These differences between resistant genotype survival show that resistance evolution is limited by emergence from the source population (i.e. *P*, *U* and *M*) and that hypermutability can ameliorate this, if only few mutations are necessary. For AB concentrations below approximately 2 × MIC_R_ survival of *N_R_* is almost 100% but drops steeply for concentrations higher than 20 × MIC where *N_R_* is replaced as the dominant genotype by the high-persister population (*P*), which stabilizes at around 35% survival for up to 50 × MIC. Other subpopulations than *N_R_* and *P* can only be found very rarely at the end of treatment, with e.g. *M_R_* surviving in approximately 2% of the treatment simulations below MIC and *U* in less than 1% in ranges where *P* populations dominate.

### (c) In contrast to resistance, persistence causes treatment failure with low numbers of surviving cells

The survival probability does not reflect the absolute pathogen load (mean number of surviving cells) of a certain genotype. For subinhibitory AB concentrations less than 0.5 × MIC, the subpopulations surviving at substantial absolute numbers are diverse (*P_R_* = 10^5^–10^8^, *M_R_* = 10^7^–10^10^, *U_R_* = 10^6^–10^8^ CFU, electronic supplementary material, figure S5). When *N_R_* populations dominate survival, they generally reach carrying capacity (10^12^ CFU). Though differing by orders of magnitude from that, the other resistant genotypes also appear at substantial numbers for AB concentrations up to MIC_R_ (*P_R_* and *U_R_* at 10^6^–10^7^ CFU) or up to 2 × MIC_R_ (*M_R_* at 10^9^ CFU) (figure 4*b*). By contrast, when *P* is the dominating population, the total population size drops drastically to less than 10 cells (figure 4*b*). These tiny populations largely consist of *P* and *U* cells (figure 4*b*), and while *U* survives considerably less frequently than *P* (figure 4*a*), in the cases where *U* survives, its cell numbers are similar to *P*. Overall, treatment failure probability above MIC is dominated by *N_R_*, with bacterial cells reaching carrying capacity, until AB doses exceed MIC_R_ substantially and only persistent, non-growing cells survive at very low numbers. These results do not change if resistant cells are allowed to switch into a persister state as well (electronic supplementary material, figure S2).

### (d) Differential genotype fitness during treatment is explained by mutational costs and persistence switching rates

To formally understand the change from *N_R_* to *P* as the dominant genotype at high AB concentrations, as well as the low probability of survival of other genotypes, we determine approximate fitness measures for all genotypes as the net growth rate far from carrying capacity (Methods). Since persisters do not grow and arise from a phenotypic—not a genetic—state change, we consider the growing and the non-growing state of a genotype together to calculate its fitness (Methods; electronic supplementary material, text S5 and S6). For AB concentrations less than 0.5 × MIC, we find the highest fitness for the susceptible genotypes *N* and *P* (figure 4*c*), as the fitness of all other genotypes is reduced by mutational costs, which outweigh the mutational benefits. However, for AB concentrations between 0.5 × MIC and 2.5 × MIC_R_, the resistant genotypes *N_R_* and *P_R_* display the highest fitness, while *M_R_* and *U_R_* grow slightly slower, due to the cost of hypermutability. Notably, fitness of all genotypes declines with increasing AB concentration, but more slowly for resistant ones. From approximately 3 × MIC onwards the rate of switching back from persistence determines the fitness of non-resistant genotypes (and hence remains constant at a negative value), with *P* and *U* having higher fitness than *N* and *M*, due to their lower back-switching rates. By contrast, the resistant genotypes continue to decline with increasing AB concentration until *P* and *U* have the highest fitness (which is equal to $-\;h_{B}s_{B}=-10^{-4}$) for AB concentrations greater than 2.5 × MIC_R_. These findings agree with the stochastic simulations of genotype survival and abundance (figure 4*a,c*), and arise from the costs of specific mutations as well as the switching rates of persister phenotypes. The residual discrepancy is explained by differences in mutation rates and in the number of mutations necessary for genotypes to emerge, i.e. how fast a genotype can emerge from *N* (*N*→*M*→*M_R_* versus *N* → *M*/*P* → *U* → *U_R_*, etc.; figure 2). The importance of mutation rates is highlighted when we consider bacteriostatic ABs, which inhibit bacterial growth and therefore mutational emergence (electronic supplementary material, text S4). We find that under treatment with bacteriostatic drugs the window where resistance is the dominant survival mechanism and the survival probability of high-persisters is drastically reduced (electronic supplementary material, figure S6).

### (e) Mutator–persisters arise from hyper-mutators during treatment and from high-persisters during relapse

Even though with very low probability and numbers, cells combining hyper-mutation and high-persistence phenotypes (*U*) can survive at the end of the treatment. This could result in subsequent treatments being ineffective due to (i) the larger fraction of persistent subpopulations, or (ii) the higher mutation rates facilitating the emergence of problematic mutations like resistance, or (iii) a combination of both. Hence, the emergence of *U* deserves closer inspection, specifically, which population do they originate from, i.e. do they emerge from the fitter *P* cells or do *M* cells acquire the beneficial high-persistence mutation? By tracking the mean cumulative mutation events of either *M* or *P* populations producing *U* cells over the course of treatment, we find that, surprisingly, despite the high survival rate of *P*, contributions from *M* to *U* are higher than the contributions from *P* for all AB concentrations (electronic supplementary material, figure S7). As the cumulative contributions to *U* do not necessarily reflect the establishment of the resulting *U* population (electronic supplementary material, text S7), we ran simulations where either only *M* or only *P* could mutate to *U*. We found that the *U* population only emerges in a similar manner as before (figure 4*b*), if *M*-to-*U* mutations are allowed (electronic supplementary material, figure S8), which makes *M* cells the main source population for *U* over the course of AB treatments.

**Figure 5.**
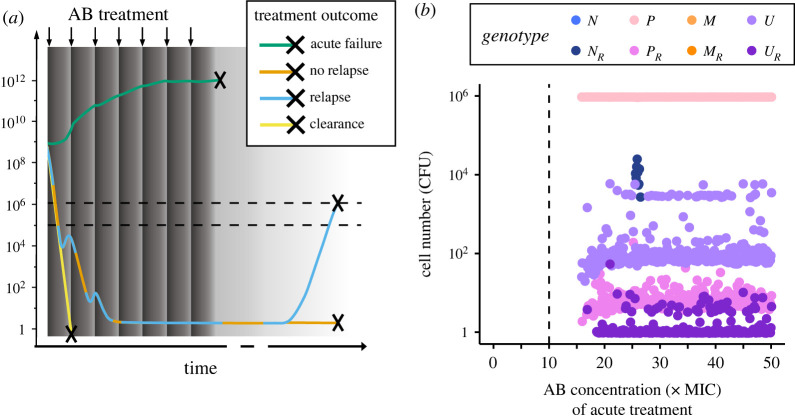
Relapse is mainly caused by persister phenotypes. (*a*) Bacteria are either cleared (yellow line) or survive 8-day AB treatment (dark grey area; periodic dosing is indicated by arrows and AB decay as a gradient). Surviving bacteria either cause immediate treatment failure (greater than 10^5^ CFU, lower dashed line) at the end of treatment (green line) or, due to regrowth from less than 10^5^ CFU, which leads either to relapse (greater than 10^6^ CFU as indicated by the upper dashed line; blue) or no relapse (remaining below 10^6^ CFU; orange) over the course of 10 years. (*b*) Mean cell numbers (CFU) of each genotype at relapse for a range of AB concentrations used in the treatment (0–50 × MIC). The vertical dashed line shows the MIC_R_.

However, *P* subpopulations are likely to become problematic after AB treatment subsides, as they provide a pool of viable cells, which can regrow in the absence of ABs and cause relapse of the infection [25]. To capture this, we model the regrowth of populations surviving 8-day AB treatment at less than 10^5^ CFU, which only occurs at AB concentrations higher than 1.5 × MIC_R_ (figure 4). Here, we assume that greater than 10^5^ cells are clinically detectable and would result in treatment continuation (figure 5*a*). More than 90% of the ‘non-detectable’ surviving populations can regrow to at least 10^6^ CFU within 10 years, causing a relapse of infection (electronic supplementary material, figure S9). The cell numbers of different genotypes at relapse, mainly correspond to *P* and *U*, as well as small *P_R_* and *U_R_* populations (figure 5*b*), suggesting that *P* and *U* subpopulations increasingly play a role in recurring infections as indicated by clinical studies [15,24]. Notably, the *N_R_* genotype only causes relapse at relatively low frequencies and for a very narrow range of AB concentrations (figure 5*b*). These populations, however, result in relapse within days (electronic supplementary material, figure S10). This is in stark contrast with the time until relapse caused by *P* and *U*, which are in a persister state at the end of treatment and must first switch back to a growing state—which on average happens within a year (median = 37 weeks; electronic supplementary material, figure S10). When considering AB treatment and relapse combined, the total contributions of *P* to *U* are higher than those from *M* (electronic supplementary material, figure S7) as *M* does not survive treatment at AB concentrations relevant for relapse simulations (figure 4*a*). Hence, while *M* is the main source population of *U* during acute infection treatment, after AB treatment ends, *P* becomes the main source population.

## 4. Discussion

Early (acute) and especially prolonged (chronic) infections with bacterial pathogens like *P. aeruginosa* or *E. coli* show a concerning frequency of mutator and high-persistence phenotypes, both of which can interfere with AB treatment success, for example by facilitating the evolution of resistance [2,5,41]. In this study, we used a stochastic population model to disentangle the complicated emergence and interplay of phenotypic and genotypic bacterial strategies that allow them to survive bactericidal AB treatment during acute infections and to establish chronic infections, i.e. relapse after treatment ends.

We find that for relatively low (but higher than MIC) AB concentrations treatment failure is certain and caused by resistant genotypes (*R*) which grow to carrying capacity by the end of the treatment (figure 4*a*,*b*). By contrast, for AB concentrations much higher than MIC_R_ treatment failure happens only in about a third of the cases and is caused by high-persistence genotypes in persister state at very low population sizes. This behaviour is explained by our deterministic fitness calculations, which show that for AB concentrations greater than 2.5 × MIC_R_ fitness of the high-persistence genotypes, *P* and *U*, is larger than that of the resistant genotypes as persisters are unaffected by high AB concentrations (figure 4*c*). *P* and *U* as the main cause for treatment failure at high AB concentrations fits with the clinically observed ‘paradox of chronic infections' [15,25], especially as peak AB concentrations reached in the treatment of cystic fibrosis (CF) patients with inhaled, nebulized ABs are high and on average greater than 2.5 × MIC_R_ [42]. Our findings suggest that treatment failure via high-persistence is most likely to occur if sufficient resistance cannot easily evolve. This means that AB concentrations targeted at exceeding MIC_R_ might instead select for ‘hidden’ infections with persisters, which can then establish chronic infections. Hence, persisters could specifically be targeted at later time points of the treatment with anti-persister drugs [43]. This strategy could be tested under laboratory conditions by treating bacterial populations with high AB doses followed by anti-persister drugs and comparing it to potential regrowth from cultures without anti-persister treatment.

In contrast with bactericidal ABs, treatment with bacteriostatic ABs inhibits cell growth but does not increase cell death. This could decelerate the emergence of resistance mutations as their emergence is contingent on bacterial growth. Indeed, when running our simulations with a bacteriostatic AB (Methods), we find that the range of AB concentrations for which resistant mutants are the predominant cause of treatment failure is smaller than for bactericidal ABs (electronic supplementary material, figure S6). Similarly, the probability of survival due to high-persisters at higher AB concentrations also decreases in comparison to treatment with bactericidal ABs due to the lower mutational emergence of *P* cells. While these results seem to suggest that bacteriostatic ABs are better at limiting bacterial survival due to resistance or persistence, they rely on the immune system to clear bacterial cells. However, many pathogens, like *P. aeruginosa*, are capable of evading the immune system, for example through quiescent cell states as those of persister cells [44,45], but also via several other mechanisms [46].

The total population size of *P* and *U* during AB treatment can only decline if the persisters switch back to the growing state, which for high-persistence mutants occurs very slowly (electronic supplementary material, figure S10), meaning that they die more slowly than other genotypes. Accordingly, we see that persister subpopulations primarily prolong the time to clearance and constitute a substantial fraction of the bacterial population only at later stages of treatment when all (or most) non-persisters have died (figure 3). The wild-type switching rates used here [17] might even underestimate persister survival as they lead to shorter lifetimes of persister subpopulations than found by Svenningsen *et al.* [47], who reported that *E. coli* persisters can survive at least 7 days of AB exposure. Using random parameter sampling and linear discriminant analysis (LDA, see electronic supplementary material, text S8 for details) to investigate the influence of transition rates between genotypic and phenotypic subpopulations (i.e. mutations and switching rates; Methods), we find that wild-type (*N*) switching rates to (*s_F_*) and from (*s_B_*) persistence influence treatment outcome substantially (electronic supplementary material, figure S11). Particularly, *s_B_* has a large impact, with higher back-switching rates leading to more clearance, which fits well with the role of *s_B_* in determining the fitness of high-persisters (figure 4*c*). This should be even more important in clinical infections, where multiple stressors are present and bacterial doubling times are generally much slower than under laboratory conditions. Yet, empirical determination of back-switching rates from persistence under different conditions and for different genotypes is scarce so far. Further, the commonly used time frames of 8–24 h might not be sufficient to empirically investigate persistence—and especially high-persistence—dynamics appropriately.

Similar to back-switching of persisters, resistance could be reversed or at least its cost compensated by additional mutations. Higher mutation rates, like in hyper-mutators, for example increase the probability for reversal of mutations, whereas compensation is more likely in wild-type cells [48]. Our simulations show, however, that the cost of resistance does not significantly influence our results and persistence remains the only escape mechanism at high AB concentrations (electronic supplementary material, figure S12).

Persistence and high-persistence have been shown to facilitate the evolution of resistance over the course of AB exposure under laboratory conditions [2,5]. Interestingly, in our simulations, *P* does not facilitate the evolution of resistance during the treatment of acute infections as illustrated by the low probability of survival of the *P_R_* genotype at the end of treatment (figure 4*a*,*b*). This is reflected in our LDA, where an extremely low percentage of simulations with random parameters result in *P_R_* as the dominant genotype (electronic supplementary material, figure S11*a*). Instead, *P* enables survival at AB concentrations where AB resistance is not viable anymore in our regimen (figure 4*c*). We find that this reasoning is robust for a wide range of parameter sets (electronic supplementary material, figure S11*c*) as higher mutation rates to resistance (*µ_R_*) and higher AB concentrations (*A*_max_) have the largest influence on pushing treatment outcome towards failure due to resistance or failure due to persistence respectively. Empirical evidence for the distinction between persistence and resistance in causing treatment failure comes from chronic *P. aeruginosa* infection in CF patients, where high-persistence phenotypes were prevalent, but only some were additionally resistant [15]. This potentially indicates that resistance via chromosomal mutations, as simulated here, might be less easily attainable or less beneficial in disease settings than in the laboratory. Additionally, the discrepancy between clinical findings and laboratory experiments regarding resistance-facilitation by persisters can partially be explained by experimental limitations: directed evolution experiments [2] use only comparatively low AB concentrations for relatively short time frames while simultaneously allowing for long AB free regrowth periods. Accordingly, while we do not find that persistence facilitates resistance evolution over the course of acute treatment, when we consider relapse after AB treatment ends, we indeed see *P*_R_ cells coming up (figure 5*b*). However, our assumption that persisters cannot mutate is likely over-simplistic and currently remains an open question in the field, but there are empirical studies indicating that persisters can still be metabolically active (reviewed in [26]) and might even increase mutation rates [49]. Further, we are not considering plasmid-borne resistance in this study, which could speed up resistance emergence, but it is unclear if plasmid conjugation occurs in persister subpopulations.

Our simulations show that, in comparison to *P*, *M* is much more likely to facilitate the evolution of resistance (figure 4), which agrees with theoretical [10] and experimental studies [11]. However, while *M_R_* readily evolves, it emerges later than *N_R_* (figure 3*c*) and grows at a slower rate due to fitness costs of hyper-mutation, which results in *M_R_* reaching lower population sizes than *N_R_*. Hence, if resistance evolves readily enough from *N*, hyper-mutators cannot dominate the population due to resistance-facilitation. However, it is still possible that *M* could acquire other beneficial mutations mitigating its cost, which are not accounted for in our model [41]. Further, in clinical conditions, *M* subpopulations might already be present at the onset of AB treatment, speeding up the emergence of *M_R_*. Nonetheless, our simulation results for *M* survival of acute treatment (figures 3 and 4*a*) are in line with hyper-mutator frequencies found in acute infections, but significantly lower than those found in chronic infections, indicating a potential role of acquiring beneficial non-resistance mutations (figure 1*a*).

In addition to the individual impact of high-persistence and hyper-mutation on the potential for treatment failure, we investigated the emergence of a combined genotype (*U*). Generally, the dynamics of the full model (figure 3*d*) mirror the dynamics of the individual sub-models for *M* and *P* regarding their survival probability and end population size (figure 3*b*,*c*). This is *a priori* not obvious for such a complicated system involving various genotypes and phenotypes and gives hope that studies of isolated systems can provide information about more complex combinations. Further, we find that *U* cells mainly emerge from the *M* population (figure 4*a*,*b*; electronic supplementary material, figure S6). Hence, in accordance with empirical studies [41], we find that *M* could hitch-hike a beneficial mutation, here the high-persistence mutation, to offset the cost of hyper-mutation. Interestingly, since the adaptive value of high-persistence comes from the non-growing persister state, the growth cost of hyper-mutation could matter less in *U*. Thus, *M* could facilitate rapid emergence of *U* early during treatment, which would allow the hyper-mutation to get fixed at minimal cost via *U*—as opposed to the situation where *M* acquires *R* and enters growth competition with other *R* genotypes (figure 3*c*).

Notably, we find that the subpopulation dynamics change between treatment failure of acute infection and relapse after treatment is discontinued. While *U* is most likely to arise from M during the treatment of acute infections, we find that during regrowth of small surviving populations, more *U* emerge from *P* than from *M* (electronic supplementary material, figure S7). Therefore, high-persistence mutants, such as *HipQ* [17,19], might not facilitate evolution on a short timescale, but rather on a longer timescale after stress subsides. Since both hyper-mutators and high-persisters are more frequent in prolonged infections, it is crucial to understand their dynamics during and following treatment (figure 5). Specifically, high-persisters have been proposed to cause recurring infections by providing a small pool of surviving cells, which start to regrow once AB concentrations subside [25]. This agrees with our findings, where relapse from small surviving populations is common (electronic supplementary material, figure S9) and predominantly caused by *P* (figures 4 and 5). Additionally, we find *P_R_*, *U* and *U_R_* genotypes in small numbers (figure 5*b*), showing that *P* does not only survive high AB concentrations to cause relapse, but also facilitates the emergence of resistance in the absence of ABs, as has been shown experimentally [2].

Our relapse model likely overestimates the time until persisters wake up in the absence of ABs, and therefore the time until relapse (electronic supplementary material, figure S10), as we assume a constant (and especially for hyper-persisters very slow) back-switching rate. This assumption corresponds to so-called ‘spontaneous persistence’ but neglects ‘triggered persistence’ [16,17], which is characterized by switching rates that are dependent on ‘trigger’ stressors, such as starvation [47] or ABs [50]. Therefore, considering triggered persistence could lead to faster switching back from persistence in the absence of ABs. Disentangling the effect and magnitude of multiple stressors on triggered switching is complicated and parameterization attempts suffer from danger of overfitting [21,51], which is likely the reason why—to our knowledge—no parameter estimates for triggered switching are available for high-persisters. Overall, our model simulations provide a conservative estimate of the probability of relapse, which could be higher with triggered persistence.

Lastly, persister frequencies show a high amount of variation in empirical studies, even in the presence of the same AB (figure 1*b*), which could be caused by stochasticity or differences in cellular physiology and persistence-causing mechanisms [52,53]. Notably, when grouped by mechanism of action, antimicrobials which target the bacterial membrane display the lowest persister frequencies [14]. All membrane-targeting antimicrobials analysed by Salcedo-Sora & Kell [14] were AMPs, which might indicate reduced persister formation or survival with AMPs as compared to ABs. Running our simulations with AMP-like pharmacodynamic parameters (Methods, electronic supplementary material, table S2), we find drastically lower survival of high-persistent and resistant bacteria (electronic supplementary material, figure S13) than for AB-like pharmacodynamics (figure 4). This is due to AMPs killing bacteria faster than ABs, allowing less opportunity for mutation emergence and switching into the persister state. This suggests that AMPs can decrease the chance of high-persister (and mutator–persister) emergence, and thereby the probability of relapse, while at the same time allowing for less resistance evolution [31].

In conclusion, we find that high-persistence and hyper-mutant genotypes mainly act independently and enable resistance evolution during different treatment stages, with hyper-mutator cells facilitating the emergence of resistance over the course of AB treatment and high-persistence after treatment ends. Accordingly, we find that the emergence of the combined mutator–persister genotype is driven by different populations during acute treatment (*M*) and during relapse (*P*). Generally, the treatment AB dose relative to the MIC of the resistant population is an important determinant for the selection of different genotypes: while genetic resistance leads to immediate treatment failure for AB levels up to 2.5 × MIC_R_, high-persistence dominates at higher AB levels, leading to relapse after drug removal. Hence, particularly the interplay of genotypes and phenotypes needs to be studied in environments with fluctuating stressors. More broadly, our modelling framework is not limited to AB treatment of bacterial infections but can be applied to other diseases, where drug efficacy is inhibited by genotypic and phenotypic mechanisms, such as in fungal infections [23,54] or cancer [55,56]. Our results suggest that treatment strategies should consider the different timescales at which various drug-escape mechanisms operate to reduce the risk of treatment failure and establishment of chronic infections.

## Data Availability

Model equations and parameters used are detailed in the manuscript and its electronic supplementary material [57]. The model code can be accessed via GitHub: https://github.com/ChrisWitzany/mutator-persister-evolution.
